# Diagnostic and prognostic value of a 7-panel mutation testing in thyroid nodules with indeterminate cytology: the SWEETMAC study

**DOI:** 10.1007/s12020-020-02411-4

**Published:** 2020-07-07

**Authors:** Stéphane Bardet, Nicolas Goardon, Justine Lequesne, Dominique Vaur, Renaud Ciappuccini, Alexandra Leconte, Hervé Monpeyssen, Virginie Saguet-Rysanek, Bénédicte Clarisse, Audrey Lasne-Cardon, Fabrice Ménégaux, Laurence Leenhardt, Camille Buffet

**Affiliations:** 1 Department of Nuclear Medicine and Thyroid Unit, Centre François Baclesse, Caen, France; 2 Department of Molecular Biology, Centre François Baclesse, Caen, France; 3 Department of Clinical Research, Centre François Baclesse, Caen, France; 4 Thyroid Unit, American Hospital, Neuilly sur Seine, France; 5 Department of Pathology, Centre François Baclesse, Caen, France; 6 Department of Head and Neck Surgery, Centre François Baclesse, Caen, France; 7 Department of Endocrine Surgery, Pitié Salpêtrière Hospital, IUC, University Paris VI, Paris, France; 8 Thyroid and Endocrine Tumors Unit, Institute of Endocrinology, Pitié Salpêtrière Hospital, IUC Sorbonne University, Paris, France

**Keywords:** Thyroid nodules, Diagnosis, Prognosis, Mutation, Thyroid cancer

## Abstract

**Purpose:**

The aim of this prospective study (ClinicalTrials.gov: NCT01880203) was to evaluate the diagnostic and prognostic value of a 7-panel mutation testing in the aspirates of thyroid nodules with indeterminate cytology (IC).

**Methods:**

Eligible patients had a thyroid nodule ≥15 mm with IC (Bethesda III–V) for which surgery had been recommended. Detection of *BRAF* and *RAS* mutations was performed using pyrosequencing and *RET/PTC* and *PAX8/PPARγ* rearrangements using Real-Time quantitative reverse transcription‐polymerase chain reaction (RT-PCR).

**Results:**

Among 131 nodules with IC, 21 (16%) were malignant including 20 differentiated cancers and one thyroid lymphoma. Molecular abnormalities were identified in 15 nodules with IC corresponding to 10 malignant and 5 benign tumours. *BRAF* mutation was detected in 4 nodules all corresponding to classic PTC, and *PAX8/PPARγ* rearrangement in 2 HCC. In contrast, *RAS* mutation was identified in eight nodules, of which four were malignant, and one *RET/PTC3* rearrangement in a follicular adenoma. This data resulted in an accuracy of 88%, sensitivity of 48%, specificity of 95%, positive-predictive value of 67%, and negative-predictive value of 91%. After a 56 month’s follow-up, the proportion of excellent response was similar in patients with molecular alterations (67%) and those without (60%).

**Conclusions:**

By increasing the overall risk of cancer from 16 to 67% in mutated nodules and by diminishing it to 9% in wild-type, this study confirms the relevance of the 7-panel mutation testing in the diagnostic of nodules with IC. Genetic testing, however, did not predict outcome in the cancer patient subgroup.

## Introduction

At least one woman out of two after 50 years old has a thyroid nodule. Owing to their frequency and the wide use of neck ultrasound (US), the clinical management of thyroid nodules has become a public health issue. Facing a thyroid nodule, the clinician has to recognize functional autonomy and to evaluate the risk of cancer. Although thyroid nodules are common, only a small fraction (~5%) corresponds to malignant tumours which are generally of good prognosis. Assessing the risk of cancer is mainly based on thyroid cytology after fine needle aspiration biopsy (FNAB) using the Bethesda classification [1]. This is a good, if not a perfect method for identifying patients with thyroid cancer. The main limitation is represented by indeterminate cytology (IC) which occurs in up to 25% of cases and does not allow differentiating between benign and malignant nodules. Indeterminate cytology comprises Bethesda class III (atypia of undetermined significance/follicular lesion of undetermined significance (AUS/FLUS)), class IV (follicular neoplasm/suspicious for follicular neoplasm (FN) or Hürthle cell neoplasm (FN/SFN)) and at a lesser degree, class V (suspicious for malignancy (SM)). Surgery is strongly recommended in Bethesda V associated with a high risk of cancer up to 75%. In clinical practice, surgery still remains a usual option for Bethesda III or IV nodules although the risk of cancer does not exceed 20–25%. As a result, ~80% of operations can be *a* posteriori considered as useless in those categories. Given the potential morbidity of thyroid surgery for parathyroid glands and recurrent nerve, and the necessity of hormonal treatment after total thyroidectomy, a more accurate preoperative assessment is needed to better select patients for surgery. This trend towards a risk-adapted approach, personalized therapy and treatment de-escalation was strengthened in the 2015 ATA guidelines [2]. Improving the characterization of nodules would allow surveillance in patients with likely benign nodules and enable a shift to the most appropriate surgical procedure in those with malignant nodules also taking into account the assumed prognosis of cancer. From this point of view, although the presence of somatic mutations, particularly *BRAF* mutation [3], has been shown to be associated with more aggressive disease, the prognostic impact of molecular testing in the aspirates of IC nodules on the long-term outcome of patients with thyroid cancer has not been evaluated so far.

In the last decade, tools of molecular analysis or imaging have been developed to improve the diagnosis of thyroid nodules. Investigating the presence of somatic mutations, genomic rearrangements or gene fusions in thyroid FNA specimens has been shown relevant in nodules with IC [4]. Testing for single mutations such as *BRAF V600E* has a high specificity for cancer but low sensitivity [5]. Testing for a limited panel of mutations (such as *BRAF, RAS, RET/PTC, PAX8/PPARγ*) enables to increase sensitivity while maintaining a good specificity [6]. More recently, ThyroSeq v2.1 next-generation sequencing (NGS) multi-gene panel of molecular markers has been shown to provide high sensitivity, 90.9 % and specificity, 92.1% in Bethesda III nodules [7]. Sophistication of ThyroSeq v3 improves sensitivity at 94.1% while moderately lowers specificity at 81.6% and enables to avoid 82% of unnecessary surgeries in patients with histologically proven benign nodules [8]. Also, recent studies in patients with IC nodules using genomic sequencing classifier have reported sensitivity ranging from 91 to 100% and specificity from 68 to 93% [9, 10].

Besides molecular testing, efforts have been made to look for imaging methods in capacity to refine diagnosis of nodules with IC. Recently, we reported the results of a prospective bicentric study designed to assess the relevance of US and shear wave elastography. Both methods failed to discriminate benign and malignant nodules [11]. The other objective of this study was to evaluate in the same patients the diagnostic and prognostic values of a 7-panel mutation testing on the indeterminate cytological specimens. We present here the results of that study.

## Patients and methods

### Patients

The study protocol was approved by the Local Ethics Committee (Ref. 2012–35, Comité de protection des personnes Nord-Ouest III) and the French Health Authorities (Ref 130213B-22). This trial is registered as ID-RCB 2012-A01313–40, ClinicalTrials.gov NCT01880203. It was conducted according to the provisions of the Declaration of Helsinki and the Good Clinical Practice Guidelines of the International Conference of Harmonization. Written informed consent was obtained from all patients.

As previously described [11], eligible patients had a thyroid nodule ≥15 mm with IC according to Bethesda classification in the six months before inclusion, for whom surgery had been recommended. Indeterminate cytology included class III, IV and V sub-categories and was confirmed by an experienced cytologist working in the other participating centre.

### Fine needle aspiration (FNA)

The FNA procedures were conducted under US guidance into the nodule of interest. The study protocol provided for two dedicated passes of FNA washed in a tube containing nucleic acid preservative solution (RNA protect^®^, QIAGEN™) which was frozen at −20 °C until analysis.

### Nucleic acids extraction

Total RNA and DNA were extracted from FNA samples using AllPrep DNA/RNA Micro Kit (QIAGEN™) according to the manufacturer’s protocol. The amount of total RNA and DNA was determined by spectrophotometry using NanoVue (GE Health Care Bio-Science, Piscataway, NJ, USA) and used as template for RT-PCR and PCR amplification.

### Detection of point mutations

DNA was amplified by polymerase chain reaction (PCR) using the following primers (*BRAF*: forward: 5′-biotin- CTTCATAATGCTTGCTCTGATAGG-3′, reverse: 5′-GGCCAAAAATTTAATCAGTGGAA-3′; *HRAS*: forward: 5′-ATTGATGGGGAGACGTGCCTGTTG-3′, reverse: 5′-biotin- TACTGGTCCCGCATGGCGCTGT-3′; *KRAS*: forward: 5′- CATGTTCTAATATAGTCACATTTTCAT-3′, reverse: 5′- biotin- AGCTGTATCGTCAAGGCACTCTT-3′; *NRAS*: forward: 5′- GCAAATACACAGAGGAAGCCTTCG-3′, reverse: 5′-biotin-GGCCAAAAATTTAATCAGTGGAA-3′;) with a product size of 226 bp, 77 bp, 121 pb and 137 bp respectively. DNA was amplified using the Quantitec Multiplex PCR NoROX kit (QIAGEN™) according to the manufacturer’s protocol. The cycling conditions were: 1- for *BRAF* and *KRAS*: 95 °C for 15 min, 45 cycles of 95 °C for 20 s, 60 °C for 30 s and 72 °C for 30 s, final extension at 72 °C for 20 min; 2- for *NRAS*: 95 °C for 10 min, 30 cycles of 94 °C for 20 s, 70°C for 20 s with a decrease of 0.5 °C per cycle and 72 °C for 45 s, 19 cycles of 94 °C for 20 s, 50°C for 20 s and 72 °C for 45 s, final extension at 72 °C for 10 min; 3- for *HRAS*: 95 °C for 10 min, 40 cycles of 95 °C for 30 s, 69 °C for 45 s and 72 °C for 30 s, final extension at 72 °C for 10 min.

Mutation detection of *BRAF* codons 600 and 601 (sequencing primer: 5′-CCACTCCATCGAGATT-3′), *HRAS* codon 61 (sequencing primer: 5′-TCCTGGATACCGCCG-3′), *KRAS* codons 12 and 13 (sequencing primer: 5′-CTTGTGGTAGTTGGAGCT-3′), *NRAS* codon 61 (sequencing primer: 5′-GACATACTGGATACAGCT-3′) using the Pyrosequencing PyroMark™ Q24 system was done following the manufacturer’s instructions.

### Detection of rearrangements

Real-time quantitative reverse transcription‐polymerase chain reaction (RT-PCR) mixture was prepared using 40 nM of each primer set and probes as previously described [12], 2X Quantitec Probe RT-PCR master Mix (QIAGEN™), Quantitec RT Mix (QIAGEN™) and 10 ng of RNA in a final reaction volume of 25 µL according to the manufacturer’s protocol. Reverse transcription two-step PCR thermal cycling for cDNA amplification and real-time data acquisition were performed with a 7500 FAST (Thermofisher™) Real-Time PCR System using the following cycle conditions: a reverse transcription step of 50 °C for 30 min, a cDNA denaturation step of 95 °C for 15 min followed by 50 cycles amplification of 94 °C for 15 s and 60 °C for 1 min. Negative control (no cDNA) and positive controls (RNA from tumours or cell lines known to carry a particular rearrangement was used as a positive control.) were cycled in parallel with each run. To decrease the likelihood of false negatives, GAPDH was amplified in parallel for each sample. Fluorescence data were analysed by the 7500 Fast Dx software and expressed as Ct, the number of cycles needed to generate a fluorescent signal above a predefined threshold. Baseline and threshold values were set by the 7500 Fast Dx software. Samples with a delta Ct inferior to 10 have been considered as positive.

### Surgery and histological examination

Thyroid surgery was performed in each participating institution and consisted in either lobectomy or total thyroidectomy. The surgeon oriented the resected specimen and localized the nodule for pathological diagnosis with the support of the descriptive diagram. The 2004 World Health Organization criteria were used for diagnosis [12].

### Initial treatment and follow-up for patients with differentiated thyroid cancer

The initial treatment for patients with differentiated thyroid cancer (DTC) was a combination of thyroid surgery, with or without neck dissection, and treatment with radioactive iodine (RAI). This treatment was discussed in a multidisciplinary team and was not affected by the presence or absence of molecular markers in FNAB samples.

After initial treatment, patients were assessed at 9–12 months and then annually. The response to treatment was evaluated according to the ATA 2015 guidelines [2]. Excellent response was defined by negative imaging and either suppressed thyroglobulin (Tg) <0.2 ng/mL or TSH-stimulated Tg <1 ng/mL, biochemical incomplete response by negative imaging and suppressed Tg ≥1 ng/mL or stimulated Tg ≥10 ng/mL or rising anti-Tg antibodies (TgAb) levels, structural incomplete response by structural or functional evidence of disease with any Tg level, with or without TgAb, and indeterminate response by nonspecific findings on imaging studies, with non-stimulated Tg between 0.2 and 1 ng/mL or stimulated Tg between 1 and 10 ng/mL, or TgAb stable or declining in the absence of structural or functional disease.

### Statistical analysis

Patient characteristics and patient subgroups were compared using the Wilcoxon or Kruskal-Wallis test (continuous variables) and chi-square or Fisher’s exact test (nominal variables), as appropriate. All tests were two-sided, and a *p* value < 0.05 was considered statistically significant. Analyses were performed with R (version 3.4.0). Sensitivity, specificity, positive predictive value (PPV), negative predictive value (NPV) and accuracy were computed to assess the diagnostic performances of the 7-panel testing. As an exploratory analysis, the link between diagnostic performances and cancer prevalence, PTC rate and *BRAF* mutated PTC rate in previous studies and ours was assessed through a linear model.

## Results

### Patient characteristics

As previously described [11], 140 patients were initially enroled in this study. Since nine patients were secondarily excluded (consent withdrawal, *n* = 4; spontaneous nodule shrinkage, *n* = 1, surgery cancelled for comorbidities, *n* = 1; surgery performed outside the participating centres, *n* = 3), 131 patients were assessable.

### Cytological and pathological data

**Fig. 1:**
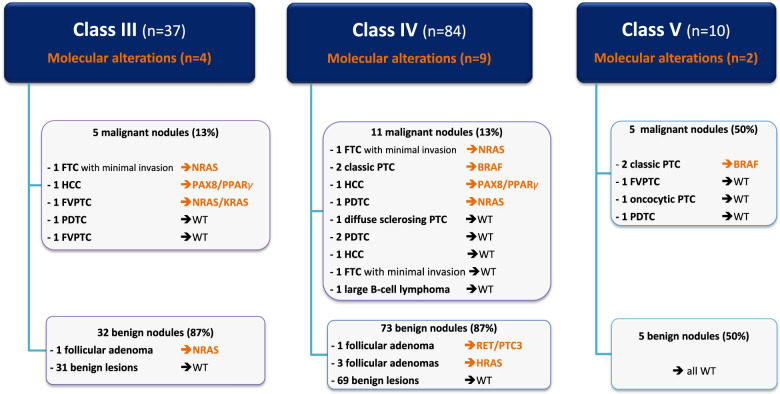
Molecular alterations in patients with malignant or benign nodules for each cytological subgroup (Bethesda III, IV and V). WT wild-type, PTC papillary thyroid cancer, FVPTC papillary thyroid cancer with follicular variant, FTC follicular thyroid carcinoma, HCC Hürthle-cell carcinoma, PDTC poorly differentiated thyroid carcinoma

From a cytological point of view, 37 patients (28%) had a nodule scored class III, 84 (64%) class IV and 10 (8%) class V (Fig. 1). Central review confirmed the IC status in all 131 nodules.

Among 131 nodules, 21 (16%) were pathologically confirmed as malignant and 110 were benign. Malignant nodules included 9 PTC (classic variant, *n* = 4; follicular variant [FVPTC], *n* = 3; oncocytic variant, *n* = 1; diffuse sclerosing variant, *n* = 1), 3 follicular thyroid carcinomas (FTC) with minimal invasion, 3 Hürthle-cell carcinoma (HCC), 5 poorly differentiated carcinomas (PDTC) and one large B-cell lymphoma. In the classes III, IV and V, the rates of cancer were 13%, 13% and 50%, respectively. Benign nodules included 61 follicular adenomas, 26 nodular hyperplasia, 15 oncocytic adenomas, 6 other pathological diagnoses (1 multinodular goiter, 1 Grave’s disease, 1 trabecular hyalinized adenoma, 3 lymphocytic thyroiditis) and 2 tumours of uncertain malignant potential. The median size of the 131 nodules was 30 mm (range, 15–71). According to EU-TIRADS classification [13], there were 52 (40%) nodules scored Tirads 3, 33 (25%) Tirads 4 and 46 (35%) Tirads 5 with no significant differences between malignant and benign nodules as previously reported [11].

### Molecular analysis on cytological specimens

Figure 1 shows the molecular alterations in patients with malignant or benign nodules for each cytological subgroup. Molecular abnormalities were identified in 15/131 nodules including 4 *BRAF* mutations, 8 *RAS* mutations (4 *NRAS*, 3 *HRAS*, 1 with both *KRAS* and *NRAS* mutations), 1 *RET/PTC3* rearrangement and 2 *PAX8/PPARγ* rearrangements. Ten (47.6%) of the 21 malignant nodules presented molecular abnormalities vs 5 (4.5%) of the 110 benign nodules (*p* < 0.001). *BRAF* mutation was identified in 4 nodules all corresponding to classic PTC. *PAX8/PPARγ* rearrangements were also associated with an HCC in the two patients where they were found. In contrast, RAS mutation was detected in 8 nodules, among which 4 were malignant (2 FTC, 1 FVPTC and 1 PDTC). Last, one *RET/PTC3* rearrangement was detected in a follicular adenoma.

Overall, molecular analysis had an accuracy of 88%, a sensitivity of 48%, a specificity of 95%, a PPV of 67% and a NPV of 91% (Table 1). Therefore, while the probability of cancer before testing in nodules with IC was 16%, the probability after testing increased to 67% in mutated-nodules and decreased to 9% in wild-type ones. There was no significant difference between the Bethesda III, IV and V subgroups in terms of accuracy, sensitivity, specificity, PPV or NPV.

**Table 1:** Test performances in the whole cohort of patients, and in the Besthesda III, IV and V groups

	All		Bethesda III		Bethesda IV		Bethesda V	
	*n* = 131		*n* = 37		*n* = 84		*n* = 10	
	Estimate	95% CI	Estimate	95% CI	Estimate	95% CI	Estimate	95% CI
True prevalence	16	[10–23]	14	[5–29]	13	[7–22]	50	[19–81]
Sensitivity	48	[26–70]	60	[15–95]	45	[17–77]	40	[5–85]
Specificity	95	[90–99]	97	[84–100]	95	[87–98]	100	[48–100]
Positive predictive value (PPV)	67	[38–88]	75	[19–99]	56	[21–86]	100	[16–100]
Negative predictive value (NPV)	91	[84–95]	94	[80–99]	92	[83–97]	62	[24–91]
Accuracy	88	[81–93]	92	[78–98]	88	[79–94]	70	[35–93]

### Outcome of patients with thyroid cancer

The patient with thyroid lymphoma and a patient with an *NRAS*-mutated FTC who was long-lost after initial treatment were excluded from the prognostic analysis. Of the 19 remaining DTC patients, 9 had a mutated tumour and 10 a wild-type tumour (Table 2). At 9–12 months after initial therapy, a similar proportion of patients with mutated and wild-type cancers presented excellent response (55% (5/9) vs 40% (4/10); *p* = 0.66). Three of 19 DTC patients received additional treatments because of persistent or recurrent disease, two with mutated tumour and one with wild-type. At last visit, after a median follow-up of 56 months (16–81), the proportion of excellent response was similar in patients with molecular alterations and those without (67% (6/9) vs 60% (6/10); *p* = 1).

**Table 2:** Outcome of patients with malignant nodules

Age (Yr)	Sex (F/M)	Cytology Bethesda	Pathology	Mutation/ Rearrangement	Date of surgery	Initial treatment (surgery, RAI)	pTNM	ATA status at 9–12 months	Additional treatment (surgery, RAI, other)	Date of last visit	ATA status at last visit
60	F	III	HCC	PAX8/PPARγ	Jul-2015	TT, RAI	pT2NxM0	ER	None	Jan-2020	ER
32	F	III	FVPTC	NRAS/KRAS	Feb-2015	TT + CND, RAI	pT1bN1aM0	IR	None	Jun-2017	ER
54	F	IV	HCC	PAX8/PPARγ	Dec-2013	TT (2), RAI	pT3NxM0	ER	None	Feb-2020	ER
49	F	IV	Classic PTC	BRAF	Jan-2014	TT (2), RAI	pT2mNxM0	ER	None	May-2019	ER
64	F	IV	PDTC	NRAS	Jan-2015	TT (2), RAI	pT3mNxM0	IR	−1st reintervention for LN recurrence in Jan-2018–2nd reintervention for LN and subcutaneous recurrence in Jan-2020	Feb-2020	SIR
42	F	IV	Classic PTC	BRAF	May-2015	TT + CND	pT1bN0M0	ER	None	Dec-2019	IR
37	F	IV	FTC minimal invasion	NRAS	Sep-2015	TT (2), RAI	pT3NxM0	ER	None	Oct-2018	ER
51	M	V	Classic PTC	BRAF	Oct-2013	TT + CND, RAI	pT4aN1aM0	SIR	Neck and mediastinum external irradiation in Jun-2014	Feb-2020	ER
49	F	V	Classic PTC	BRAF	Feb-2014	TT(2) + CND, RAI	pT2 N1a M0	IR	None	Feb-2020	IR
47	F	III	PDTC	WT	Jun-2013	TT(2) + CND, RAI	pT3N0M0	ER	None	Feb-2020	ER
63	F	III	FVPTC	WT	Mar-2015	TT(2), RAI	pT2NxM0	ER	None	May-2019	ER
39	F	IV	Diffuse sclerosing PTC	WT	Jun-2014	TT + CND, RAI	pT3N1aM0	IR	None	Mar-2019	ER
68	M	IV	PDTC	WT	Mar-2014	TT, RAI	pT3NxM0	IR	2nd RAI treatment for Tg increase in Dec-2019	Dec-2019	SIR
83	M	IV	PDTC	WT	Mar-2014	TT (2), RAI	pT2NxM0	ER	None	Feb-2020	ER
36	F	IV	HCC	WT	Mar-2015	TT(2) + CND	pT2N0M0	ER	None	Mar-2019	ER
36	F	IV	FTC minimal invasion	WT	Jun-2015	TT(2), RAI	pT2NxM0	IR	None	Mar-2020	ER
76	F	V	FVPTC	WT	Jan-2014	TT	pT2NxM0	IR	None	Apr-2018	IR
59	F	V	Oncocytic PTC	WT	Jul-2014	TT	pT1aN0M0	IR	None	Oct-2015	IR
80	F	V	PDTC	WT	Oct-2014	TT + CND, RAI	pT3N0M0	BIR	None	Dec-2017	Death^a^ BIR

*WT* wild-type, *PTC* papillary thyroid cancer, *FVPTC* PTC with follicular variant, *FTC* follicular thyroid carcinoma, *HCC* Hürthle-cell carcinoma, *PDTC* poorly differentiated thyroid carcinoma, *TT* total thyroidectomy, *TT (2)* total thyroidectomy in two times, *CND* central neck dissection, *RAI* radioiodine treatment, *ER* excellent response, *IR* indeterminate response, *BIR* biochemical incomplete response, *SIR* structural incomplete response

^a^The patient died of metastatic colic carcinoma and presented with evidence of BIR just before

### Comparison of present data with previous studies

**Fig. 2:**
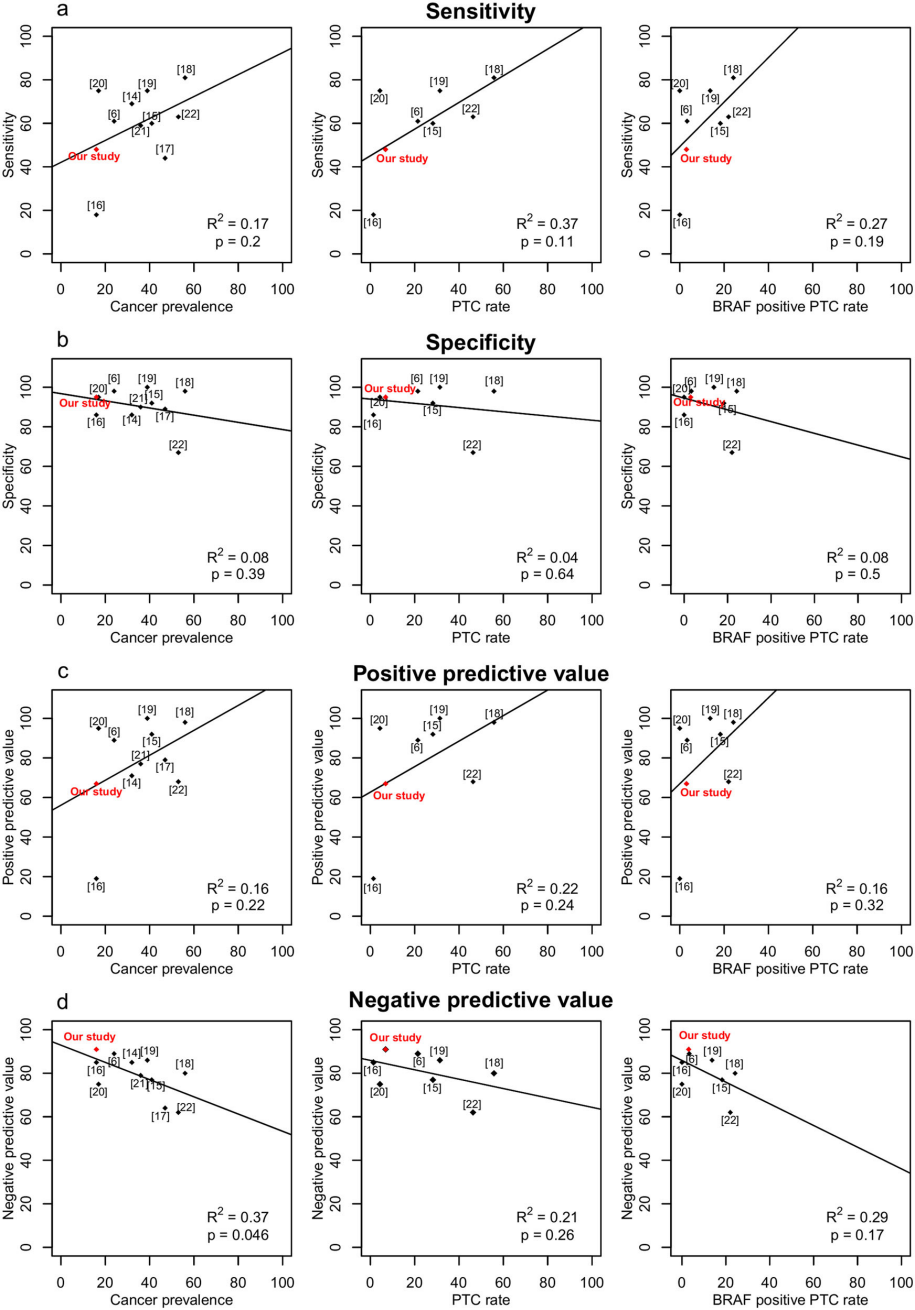
Test performances (**a**, sensitivity; **b**, specificity; **c**, PPV; **d**, NPV) according to cancer prevalence, PTC rate and *BRAF* positive PTC rate in previous studies (6, 12, 14–20) and in the present study. The quality of the linear model adjustment is displayed on each graph (*R*^2^ and *p* value)

The analysis of the present data with previously reported studies [6, 14–22] is shown in Table 3 and in Fig. 2. In each study, we extracted the nodules with AUS/FLUS, FN/SFN or SM cytology, or Bethesda classes III, IV and V, or with “indeterminate” cytology. Only nodules operated on were taken into account for analysis. The number of cases in each study varied from 23 to 513, cancer prevalence from 16 to 56%, PTC rate from 1 to 56% and rate of *BRAF* positive PTC tumours from 0 to 24%. Sensitivity ranged from 18 to 81%, specificity from 86 to 100%, PPV from 19 to 100% and NPV from 64 to 91%. The sensitivity (48%) and PPV (67%) estimated in our series were generally lower than in other studies while NPV (91%) was slightly higher.

**Table 3:** Analysis of the present data in comparison with that of the literature

Reference 1st author, yr, [ref. number]	Cytology (*n* in each subcategory)	Operated nodules, *n*	Malignant nodules, *n* (%)	PTC, *n* (%)	BRAF positive PTC, *n* (%)	Test performance, %			
						Se.	Sp.	PPV	NPV
Nikiforov 2009 [19]	FLUS (21), FN (23), SM (7)	51	20 (39%)	16 (31%)	7 (14%)	75	100	100	86
Cantara 2010 [18]	Indeterminate (41), SM (54)	95	53 (56%)	53 (56%)	23 (24%)	81	98	98	80
Nikiforov 2011 [6]	AUS/FLUS (247), FN/SFN (214), SM (52)	513	121 (24%)	110 (21%)	17 (3%)	61	98	89	89
Beaudenon-Huibregtse 2014 [17]	AUS/FLUS (22), FN/SFN (19), SM (12)	53	25 (47%)	na	na	44	89	79	64
Eszlinger 2014 [16]	Indeterminate	141	22 (16%)	2 (1%)	0 (0%)	18	86	19	85
Eszlinger 2015 [15]	Thy 3 (163), Thy 4 (39)	202	83 (41%)	57 (28%)	37 (18%)	60	92	92	77
Labourier 2015 [14]	III (58) IV (51)	109	35 (32%)	na	na	69	86	71	85
Bongiovanni 2015 [20]	FN/SFN (23)	23	4 (17%)	1 (4%)	0 (0%)	75	95	95	75
Mancini 2012 [21]	Thy 3 (38), Thy 4 (9)	47	17 (36%)	na	na	59	90	77	79
Bellevicine 2020 [22]	AUS/FLUS (86), FN/SFN (34), SM (57)	177	93 (53%)	82 (46%)	39 (48%)	63	67	68	62
Present study	III (37), IV (84), V (10)	131	21 (16%)	9 (7%)	4 (3%)	48	95	67	91

III, IV, V: for Bethesda class III, IV or V

Thy 3, equivalent to FN/SFN; Thy 4, equivalent to SM

*AUS/FLUS* atypia of undetermined significance/follicular lesion of undetermined significance, *FN/SFN* follicular neoplasm/suspicious for follicular neoplasm (FN) or Hürthle cell neoplasm, *SM* suspicious for malignancy

Although not significant, data shows a trend to a link between diagnostic performances of genetic testing and cancer prevalence, PTC rate and proportion of PTC harbouring *BRAF* mutations in each cohort, especially regarding sensitivity and PPV (Fig. 2).

## Discussion

This prospective bicentric study confirms the clinical relevance of a 7-panel mutational testing in the characterization of cytologically indeterminate nodules. Molecular testing shows a good specifity (95%), a more limited sensitivity (48%) and an acceptable accuracy (88%). In this series of patients with a 16% pre-test probability of cancer, a 67% PPV and 91% NPV mean that the post-test probability increased to 67% in mutated-nodules and decreased to 9% in wild-type ones.

The impact of a 7-panel mutational testing on the diagnosis of IC nodules has been reported in previous studies [6, 14–22]. As shown in Fig. 2, the analysis of the present data and the literature shows that our sensitivity (48%) and PPV (67%) was generally lower than in previous studies while NPV (91%) was moderately higher. These studies were conducted between 2009 and 2018, and included nodules with variable proportions of AUS/FLUS, FN/SFN or SM cytology, using Bethesda classification or not, resulting in a wide range of cancer prevalence from 16 to 56%. Similarly, the PTC rates were highly variable ranging from 1 to 56% as well as the proportions of *BRAF* mutated PTC tumours from 0 to 24%. Given its frequency, the presence of *BRAF* mutations significantly affects the results of the 7-panel testing in IC nodules. *BRAF* mutation is characteristic of PTC but only a part of them (45 to 80%) are *BRAF* mutated. This variable proportion is linked to pathological variants [23], classic PTC tumours being more often *BRAF* positive than other PTC variants, and to the geographical origin of patients with Koreans showing very high proportions of *BRAF* mutated PTC tumours [24]. The quite low sensitivity observed in the present study (48%) can be explained by a 16% cancer prevalence and a 7% PTC rate at the low end of the expected range. Nevertheless, the histological distribution of the study group is consistent with what we could expect from IC, namely a combination of classic PTC (19%), FVPTC (14%), FTC (14%), and HCC (14%) and PDTC (24%). As cancer prevalence also impacts NPV and PPV [25], this could have resulted in “underestimating” a 67% PPV and “overestimating” a 91% NPV.

The prevalence of *PAX8/PPARγ* rearrangements is generally limited in IC nodules with no cases reported in some studies [15, 18] and only one in others [17, 19]. Our data confirms that *PAX8/PPARγ* rearrangements are cancer specific. Nevertheless, whereas *PAX8/PPARγ* rearrangements were identified in three FVPTC and one FTC in Nikiforov’s study [6], in our series there were associated with two patients with HCC. The findings in the three HCC of our cohort (i.e., two cases with *PAX8/PPARγ* rearrangements and one wild-type tumour) were quite unexpected. Indeed, the prevalence of *PAX8/PPARγ* rearrangements is generally low in HCC, estimated at 5% in the review by Maximo et al [26] although rates up to 27% have already been reported [27]. The association between *RET/PTC* rearrangements and HCC is more prevalent and has been estimated at 35% [26]. Comprehensive analysis of the molecular landscape of HCC has very recently been achieved showing that these tumours exhibit a wide range of recurrent mutations, notably of the mitochondrial genome and high DNA copy-number alterations [28, 29].

In contrast, and as expected, *RET/PTC* rearrangements and *RAS* mutations presented more limited diagnostic values. No *RET/PTC* rearrangements were found in malignant tumours and one *RET/PTC3* was detected in a benign nodule. The presence of *RET/PTC* rearrangements is possible in benign nodules and a recent systematic review in 2239 benign lesions from 38 studies showed a prevalence of *RET/PTC* rearrangements ranging from 0% to 68% [30]. A study performed in PTC tumours also suggests that the variability in the rate of *RET/PTC* rearrangement could also be related to the use of different detection methods and tumour genetic heterogeneity [31]. With respect to RAS mutations, they were detected in eight nodules, half of them corresponding to malignant lesions (2 FTC, 1 FVPTC, 1 PDTC) leading to a 50% PPV. Although comprehensive pathological data was available in a few previous studies [6, 15, 16], true-positive *RAS* mutations were observed mainly in FVPTC or FTC, and sometimes in classic PTC, HCC or PDTC. False positives were found in all previous studies, and the estimated PPV for *RAS* testing ranged from 13 to 92% [6, 15, 16]. As in previous studies, mutations were present in follicular adenomas. No non-invasive follicular thyroid neoplasm with papillary-like nuclear features (NIFTP) was found in our series. Recently, it has been reported that a substantial proportion (47%) of NIFTP could harbour *NRAS* mutations [32].

**Fig. 3:**
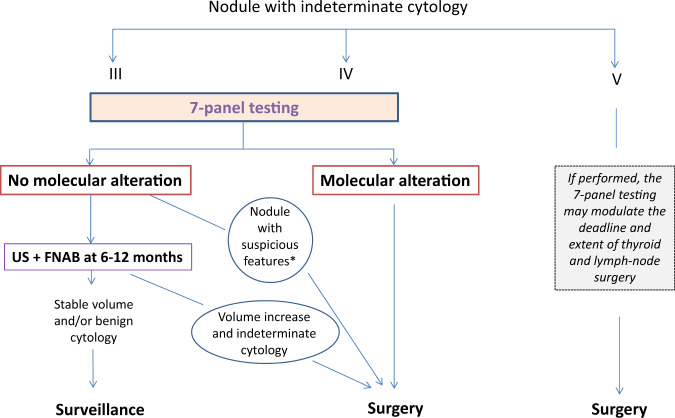
Proposal for the use of 7-panel testing to guide clinical decision in patients with cytologically indeterminate nodules

Based on our data and that of literature, a flow chart for the use of 7-panel testing to guide clinical decision in patients with IC nodules is proposed in Fig. 3. Given the generally high risk of cancer in Bethesda V nodules, and limited sensitivity of the 7-panel testing, surgery is recommended in all Bethesda V patients. If performed, genetic testing may modulate the extent of thyroid and lymph-node surgery. The similar performances of the 7-panel testing in Bethesda III and IV nodules suggest using it in both categories of patients. Surgery appears to be recommended when a molecular alteration is detected, particularly *BRAF* or *PAX8/PPARγ* which are highly specific for cancer. In the absence of molecular alteration, a US and FNAB control at 6–12 months could be performed, except for nodules suspicious of cancer for other reasons, e.g., history of radiation, serum calcitonin increase or EU-Tirads 5, which should be operated on. The absence of volume progression at 1 year, all the more if associated with a benign cytology, would allow continuing spaced monitoring.

An issue that has not been resolved to date is whether 7-panel molecular testing of cytology specimens could help predict long-term outcomes in the cancer patient subgroup. Our data show that the outcome of patients with or without molecular alterations was not different, suggesting that mutational testing in FNAB with 7-gene panel may play a minimal role in identifying high-risk cancers. Again, since the *BRAF* mutation is the most common alteration associated with aggressive behaviour in PTC [3], the fairly low rates of PTC and *BRAF*-mutated PTC in our series may have contributed to such negative results. Above all, there is evidence pointing out that the presence of multiple molecular alterations or mutations such as those of *TERT* [33], *TP53* or *PIK3CA* [34, 35], that are not included in the 7-gene panel, have a higher prognostic value. In any event, this prognostic analysis has been performed in a limited number of patients and must be confirmed in larger series.

The strengths of the present study are its prospective design, the confirmation of IC by an independent review, the histological gold standard and the ability to assess the prognostic value of the mutational testing with a significant follow-up. The study also presents some limitations. One is that certain mutations of the *RAS* genes, notably *HRAS* codon 13 mutations previously reported in anaplastic thyroid cancer, and *KRAS* codon 61 in both PTC and PDTC, were not analyzed. This may have underestimated the number of positive cases although these mutations are uncommon [35]. Above all, a limitation was not having access to NGS multi-gene panel such as ThyroSeq v3 [36] or other recently reported technologies [37–39]. The use of multi-gene NGS panels makes it possible to analyse more than one hundred genes and to detect different classes of genetic alterations, including mutations, insertions and deletions, gene fusions, gene expression alterations and copy number variations to improve diagnostic accuracy and potentially prognostic value of genetic testing on cytologically indeterminate nodules. When the study was launched, the NGS multi-gene panel was not available. The cost of NGS panels remains a critical problem, raised in the same way by the genomic tests currently available. In countries or institutions subject to financial constraints, and before the widespread use of NGS technics, this would support a relevant role in clinical practice for a small panel of genes.

In conclusion, this prospective study confirms that the 7-panel mutation testing is a simple and low-cost tool to help the clinician manage patients with cytologically indeterminate nodules. In the subgroup of cancer patients, however, mutational testing has not been shown to have a significant prognostic value.
